# Digital Medication Adherence Support: Could Healthcare Providers Recommend Mobile Health Apps?

**DOI:** 10.3389/fmedt.2020.616242

**Published:** 2021-02-17

**Authors:** Claudine Backes, Carla Moyano, Camille Rimaud, Christine Bienvenu, Marie P. Schneider

**Affiliations:** ^1^Lab of Medication Adherence and Interprofessionality, School of Pharmaceutical Sciences, University of Geneva, Geneva, Switzerland; ^2^Institute of Pharmaceutical Sciences of Western Switzerland, University of Geneva, Geneva, Switzerland; ^3^Center for Primary Care and Public Health (Unisanté), University of Lausanne, Lausanne, Switzerland; ^4^Pharma24, An Academic Community Pharmacy and Living Lab Located at the Exit of the Geneva University Hospitals, Geneva, Switzerland

**Keywords:** medication adherence, mHealth, eHealth, chronic diseases, pharmacists, app evaluation, healthcare provider

## Abstract

Adherence to prescribed medication is suboptimal in 50% of the chronic population, resulting in negative medical and economic outcomes. With the widespread use of mobile phones worldwide, medication adherence apps for mobile phones become promising medication adherence aids thanks to simplicity, user-friendliness, and accessibility for the public. Yet, until today, there is insufficient evidence in favor of using mobile health (mHealth) apps to increase medication adherence. This study aims to develop a methodology for scientific and end-user (patient) mHealth evaluation (a) to identify medication adherence apps search terms, (b) to evaluate identified apps based on scientific criteria, and (c) to report best smartphone apps evaluated by patients. Search terms were identified *via* literature review and expertise. Firstly, an online questionnaire was developed to identify frequently used search terms by recruited patients. Related medication adherence apps were identified and selected using predefined inclusion criteria. Secondly, identified apps were evaluated thanks to a scientific evaluation method and a created online questionnaire for patient feedback. Recruited patients were invited to test and evaluate the selected apps. Out of 1,833 free-of-charge and 307 paid apps identified, only four free-of-charge and three paid apps remained included in the study after eligibility criteria. None of the selected app reached a high score. Looking at the overall scores, Medisafe (59%), MyTherapy (56%), and Meds on time (44%) received the highest scores in the scientific app evaluation. In the patient evaluation, Dosecast (3.83 out of five points), Medisafe (3.62), and SwissMeds (3.50) received the highest scores. None of the apps in this research has undergone a process for certification, for example, CE marking, through a notified body. Security and data protection aspects of existing apps highly contribute to these low evaluation scores through little information on patient's data processing and storage. This might be corrected through the introduction of General Data Protection Regulation (GDPR) in the European Economic Area (EEA) and more scrutiny through regulatory bodies in the EU/EEA and the USA. None of the applications should be recommended by healthcare providers. In addition, clinical studies with chronic patients are necessary to measure long-term app impacts.

## Introduction

Medication adherence is highly associated with healthcare clinical outcomes such as treatment effectiveness, medical visits, hospitalization, and morbidity and mortality rates. This is especially true when it comes to chronic disease treatments (1–4). Optimal medication adherence rates bring benefit to both patients and the healthcare system (5). The World Health Organization (WHO) reports 50% of the population being non-adherent, resulting in negative medical and economic outcomes. This percentage is reportedly increasing in parallel with the rise in the prevalence of chronic diseases worldwide (6–9). Thanks to scientific innovation, numerous medication plans have changed from acute to chronic, increasing life expectancies, quality of life, patient autonomy, and self-determination. To improve patients' long-term adequate drug management, healthcare providers need to partner with patients to define clear treatment objectives and to reinforce patients' autonomous and active role in adhering to their treatment plans.

Numerous initiatives aim to support patients' medication adherence by targeting the four identified major influencing factors: (i) education, (ii) behavior, (iii) cognitive behavior, and (iv) multi-approach (10–14). Recent studies underline high adherence rates in conjunction with improved interprofessional communication and collaboration of the medical sector (15, 16). Defined as the process of a patient's intake of prescribed treatments, medication adherence includes the three phases of treatment initiation, implementation, and persistence (17). “Implementation” describes the daily drug intake during the time span of “persistence,” which is defined as the duration between treatment “initiation” and “discontinuation.” To correlate medication adherence with clinical outcomes, measurement methods are necessary (9). In the literature, indirect measures such as electronic monitoring are reported with high validity (18). Simultaneously, aiming to increase medication adherence, different tools are being developed in the digital health (or eHealth) sector with constantly growing profile and use.

Mobile health (mHealth) is an integral part of the digital health sector and is defined by the WHO as a healthcare and public health service delivery supported by digital health tools on mobile devices (19). Health applications (apps), also described as mHealth software, deliver health information and communication technologies in support of health fields, collect healthcare data, and may be connected to wearables, also described as mHealth hardware (20). While digital technologies are becoming important resources for the entire healthcare sector, mobile wireless technologies are particularly relevant, thanks to their user-friendliness, broad reach, cost-effectiveness, and popular public acceptance. In 2018, according to the International Telecommunication Union (ITU), 5.3 billion active mobile-broadband phone subscriptions were in use globally, demonstrating sustained growth (21). On the market for 10 years, mHealth apps have steadily increased with 78,000 new health apps added to major app stores in 1 year (2017) resulting in a total of 325,000 mHealth apps available in 2017. Of those, 10,000 apps focused on medication reminders (22, 23).

If an mHealth app is able to increase medication adherence, it has promising potential to improve a patient's quality of life and life expectancy while also monitoring healthcare cost (24). However, patients' data collection and related GDPR issues may limit such an app's usability. Sensitive personalized data may be transferred to third parties, be modified, or even get lost. Further, the quality of the medical information may be poor or misleading (25). To tackle these issues, medical regulations and guidelines (26, 27), data protection regulations (28), and national (29) and European (20) recommendations have been developed.

Until today, there is insufficient evidence in favor of using mHealth apps (30) although, in 2015, a review identified a positive impact on medication adherence through mobile phone applications (31). For mHealth to be adopted as part of routine clinical practice, collaboration with healthcare practitioners is essential. To encourage patient use of mHealth technologies, healthcare providers need to understand and recommend adapted technologies. However, a recent study reported that healthcare providers have high concerns: first, about digital health technologies' lack of official approval from government or similar regulatory bodies and, second, about the sparse studies demonstrating safety or effectiveness of such technology (32).

This study aims to propose a novel methodology enabling (a) to identify search terms and smartphone applications for medication adherence of chronic diseases, (b) to evaluate identified apps and establish a ranking based on scientific criteria, and (c) to report the best smartphone applications evaluated by patients. The results of the study should support healthcare providers with criteria for advising and recommending medication adherence applications.

## Materials and Methods

The study focused in a first step on free-of-charge medication adherence apps. In a second step, the methodology was replicated and adapted to paid apps. Most importantly, a method for scientific (objective) and patient evaluation (subjective) to assess medication adherence apps was developed.

### Identification of Search Terms for Medication Adherence Apps

A list of primary search terms (French or French and English) was created by establishing a preliminary list of search terms based on previously published articles (24, 33–35) and mHealth expertise (CM, MS, CB, CR). Subsequently, the list and a questionnaire were sent to patients online to identify the main search terms used by patients when searching for medication adherence apps (Appendix 2). Patients also rated how likely they would use already identified search terms.

### Smartphone Application Identification

The retained search terms allowed for the identification of medication adherence apps for smartphones in Swiss stores, specifically the Apple App Store (iOS, Apple Inc.) and Google Play Store (Google LLC.). Medication adherence app inclusion and exclusion criteria were adapted based on previously published criteria (24, 33) (Table 1). The first 50 apps occurring from each search term were included.

**Table 1:** Study inclusion and exclusion criteria for mobile medication adherence applications.

**Inclusion criteria (*n* = 5)**	**Exclusion criteria (*n* = 9)**
French or French and English	Targets specific populations
Recent updates defined as ≤18 months	Targets a specific medication
Included in both stores, Apple App Store (iOS) and Google Play Store	Targets a specific disease
No bugs during the opening of the app	Imposes medication online refill (e.g., connected to refill store)
Targets human population	Targets lifestyle, wellness, or fitness adherence
	No inclusion of medication information (e.g., adverse events) or only inclusion of general reminder functions (e.g., calendar, agenda, etc.)
	Games
	Only for training purposes
	No clinical use

### Scientific App Evaluation Criteria

A list of criteria to scientifically evaluate app quality was created based on various criteria collected from literature research (24, 36–38), national recommendations (39), and European recommendations (20). A panel of three experts and one e-patient assessed these criteria, categorizing each as “very important,” “important,” and “not important.” Only “important” and “very important” criteria were kept.

These criteria are divided into six categories: (i) security and privacy (five items), (ii) quality of the health-related content (4), (iii) quality of the app information management (6), (iv) functionality (11), (v) user interface, and (vi) acceptability of the app (Table 2). For each criterion, a definition was developed on what circumstances the scientific evaluation requirement was not met (zero point) and was met (one point). Each included application was tested for 10 days by an investigator (CM, CR) with the help of these scientific evaluation criteria.

**Table 2:** Definition of categories and point criteria for scientific evaluation of the quality of the smartphone apps.

**Rating**		**0 point**	**1 point**
**Category: security and privacy**			
1	Password	The user cannot protect access with a user name and/or password.	The user can protect access with a user name and/or password.
2	User consent	The user is not able to approve use of one's data (e.g. geographical location, calendar, and photo).	The user can approve use of one's data (e.g. geographical locations, calendar, and photo, through a notification or settings).
3	User consent revisable	The user does not have the right to modify or revoke consent while using the app.	The user has the right to modify or revoke consent any moment.
4	Erasing of user data	The app does not allow erasing of user data.	The app allows erasing the user data or gives instructions on how to proceed with erasing.
5	Data collection	The information on data collection is not available in the terms and conditions.	The information on data collection is available in the terms and conditions.
**Category: quality of the health-related content**			
6	Aim	The aim of the app is not provided in the descriptions of the store.	The aim of the application is provided in the descriptions of the store.
7	Education	Does not provide educational information.	Does provide educational information.
8	Involvement of healthcare professionals	A message that the app does not substitute healthcare professionals is not available in the app and/or terms and conditions.	A message that the app does not substitute healthcare professionals is available in the app and/or terms and conditions.
9	Trustworthiness, credibility, and quality of the educational information	Sources with little information, not traceable or not trustworthy.	Verifiable sources with high-quality, sufficient quantity, credible, and evidence-based medicine.
**Category: quality of the app information management and topic-related information**			
10	Certification (CE marking)	The application is not CE certified.	The application is CE certified.
11	Content author's expertise	The content author's expertise is not mentioned.	The content author's expertise is mentioned.
12	Evaluation by target population	The app does not mention if an evaluation by the target population has been performed.	The app mentions that an evaluation by the target population has been performed.
13	Declaration of interest	A declaration of interest is not mentioned.	A declaration of interest is mentioned.
14	Sources and references	Sources and literature references are not stated.	Sources and literature references are stated.
15	Funding	The sources of funding are not stated.	The sources of funding are stated.
**Category: functionality**			
16	Reminders: text messages and push notifications	The app does not provide a reminder function.	The app provides a reminder function.
17	Visual feedback (e.g., graphs, statistics)	The app does not provide visual feedback.	The app provides visual feedback.
18	Meetings (with healthcare professionals)	The app does not allow to arrange meetings.	The app allows to arrange meetings.
19	Cloud/synchronization of data on different devices	The data is not saved on a cloud. The users are not able to synchronize their user account across different devices.	The data is saved on a cloud. The users are able to synchronize their user account across different devices.
20	Access and sharing of different phone functions (e.g., between app and camera, calendar)	The user cannot choose to accept sharing and access to data through other apps.	The user can choose to accept sharing and access to data through other apps.
21	Patient file	No connection to the patient's medical or pharmaceutical files.	Connection possible to the patient's medical or pharmaceutical files.
22	Manual entries and comments	Comments cannot be entered in the app.	Comments can be entered in the app.
23	Gamification	The app does not advertise or send notifications for gamification.	The app advertises or sends notifications for gamification.
24	Support (e.g., hotline, FAQ, user's manual, instructions)	The app does not provide support functions.	The app provides support functions or a FAQ.
25	Refill (indication of number of remaining doses to anticipate next purchase)	The app does not contain an option for a refill reminder.	The app contains an option for a refill reminder.
26	Time zone adaptation	The app does not have an option for adaptation to new time zone.	The app provides an option for adaptation to new time zone.
**Category: esthetics**			
27	Flexibility	The user cannot choose the interface or functionalities.	The user can choose the interface layout.
28	Legibility (e.g., text, images)	The app does not provide an option to change font size.	The app provides an option to change font size.
29	Customization (e.g., design interface, alarm type)	The user cannot customize the app.	The user can customize the app.
**Category: advertising**			
30	Advertising	The app contains advertisements.	The app does not contain advertisements.

### Criteria for App Evaluation by Patients Based Upon uMARS

A new online uMARS (user version of the Mobile Application Rating Scale) questionnaire was created in French (36) (Appendix 3) and sent to recruited patients *via* Google Sheets. This questionnaire was based on various uMARS versions: an Italian version, the non-validated French version, and the original English uMARS version. The evaluation based on uMARS aims to assess the usability and suitability of the previously identified apps in chronic patients.

### Inclusion of Patients

Patients voluntarily participated in the study and were selected by defined inclusion criteria (Appendix 4). Patients having accepted replying to the questionnaire about frequently used search terms (section identification of search terms for medication adherence apps, Appendix 2) were asked to volunteer to test an app at random.

For the first step evaluation of free-of-charge apps, we included e-patients. These apps were evaluated by e-patients for 10 days. The e-patient is defined as a patient who is an expert in his disease field and highly at ease in using digital health tools and information. An e-patient is part of the investigators of the study; she is a professional patient working at a university medical institution and leading a voluntary e-patient platform, participating in various research studies.

In the second step evaluation, to increase external validity in the general population, paid apps were not tested by e-patients but by regular chronic patients recruited *via* flyers, web ads, and the Facebook page of Pharma24, the academic community pharmacy located at the exit of the University Hospitals of Geneva, Switzerland. Ethics committee approval from the canton of Geneva, Switzerland, was obtained through application 2018-01398. The University paid for the app fees. These patients then tested the applications for 14 days, with randomization of which patient was testing which app (Appendix 5).

## Results

### Search Terms Identified

A total of 147 search terms were identified. Through elimination of duplicates and terms with little relation to medication adherence, a total of 17 English and 65 French search terms have been identified for the search of medication adherence apps (Appendix 6).

The patients included in this study (*N* = 16 for free-of-charge app testing and *N* = 10 for paid app testing) were mainly between 30 and 60 years of age (*n* = 14), taking medication since more than 10 years (*n* = 8) (Table 3).

**Table 3:** Demographics of patients/e-patients that replied to the questionnaire.

**Category**		**Free-of-charge apps (*n* = 16)**	**Paid apps (*n* = 10)**
Gender	Male	6	5
	Female	10	5
Age groups	20–29	1	2
	30–39	5	2
	40–49	4	3
	50–59	5	3
	60–69	1	/
Chronic medication intake	Yes	6	10
	No	10	/
No. of chronic medication per day	1	n/a	5
	2	n/a	2
	3	n/a	1
	4	n/a	1
Types of chronic disease	Cardiovascular	2	/
	Kidney disease	2	/
	Respiratory	1	1
	Pain	1	/
	Diabetes	1	/
	Neurodegenerative	/	4
	Dermal	/	1
Medication intake history	2–5 years	1	2
	5–10 years	2	2
	More than 10 years	3	5
	Missing	10	1
Use of mHealth	Yes	10	0
	No	6	10
Use of health app	Yes	16	2
	No	0	8

**Figure 1:**
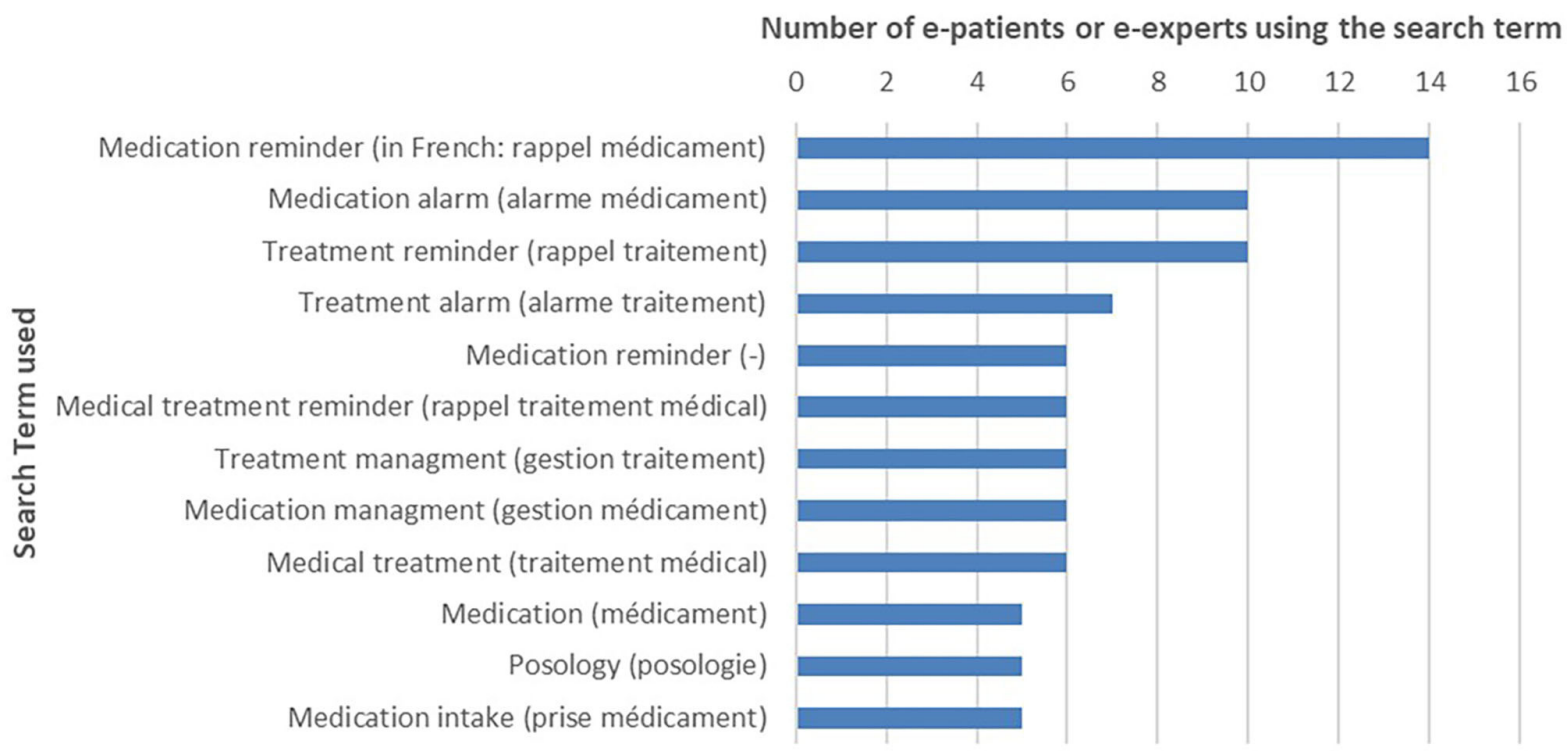
Medication adherence apps: search terms selected by e-patients (*N* = 16) and eHealth experts (search term nominated at least five times).

The search terms most likely used by patients are described in Figure 1. “Medication reminder” (French: “rappel médicament”), “medication alarm” (“alarme médicament”), and “treatment reminder” (“rappel traitement”) were mainly used. Only one English search term was selected, “medication reminder.” The search terms with at least five nominations from patients and mHealth experts have been retained for the next steps.

### Apps Meeting the Inclusion Criteria

**Figure 2:**
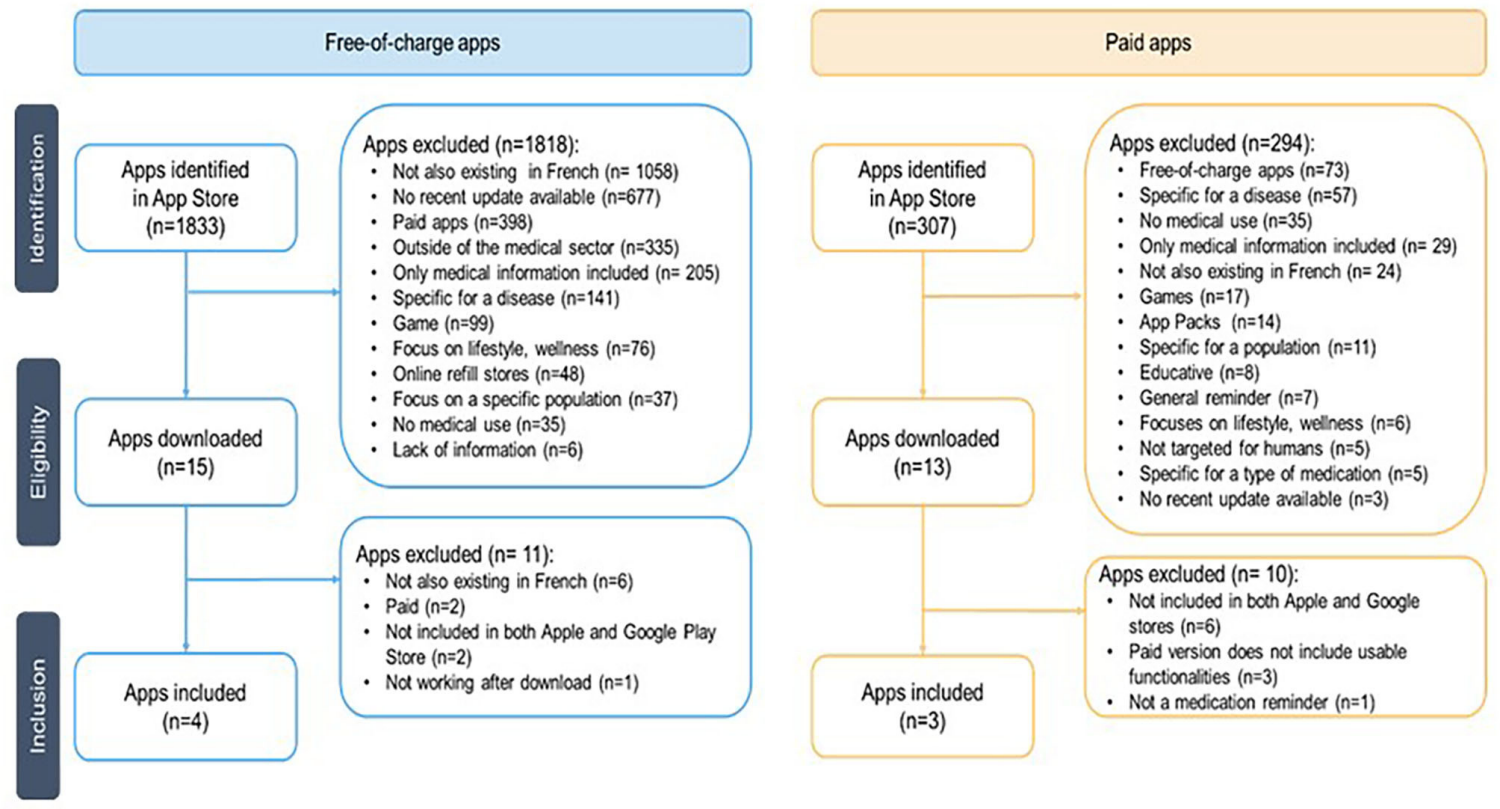
Flowchart for the selection process for free-of-charge and paid apps.

The flowchart (Figure 2) presents the systematic selection of apps included and evaluated in this study. A total of 1,833 free-of-charge apps have been identified in the Swiss Apple App Store, 567 paid apps in Google Play Store, and 307 paid apps in the Apple App Store.

Having identified these apps, the search terms for this study (Appendix 6) were revisited looking at what search terms matched the later included free-of-charge apps *Medisafe, MyTherapy, Meds on time*, and *Médi'rappel* (Appendix 1). Out of the 82 initial search terms, 26 led to the free-of-charge apps included in the study, 33 did not meet the eligibility criteria, and 23 terms did not provide any result. Additionally, *SwissMeds* (no pricing yet defined as it is currently at the end of its development stage by the Geneva University Hospitals) was added to the list.

### Evaluation of Quality of the Smartphone Apps Through Scientific Criteria and Patient Evaluation

Free-of-charge apps included are *Medisafe* (free version tested; premium version available), *MyTherapy, Meds on time*, and *Médi'rappel*. Paid apps included are *Dosecast* (pricing: service fee of 2.99 CHF/month), *Rappel de medicament* (2 CHF/download), and *Suivre ma Rx* (7 CHF/download*)*. At the time of evaluation, SwissMeds was not yet available in the app stores as it was only made available for testing as a prototype, and yet, it fulfilled all the inclusion criteria described in Table 1.

#### Scientific Evaluation Based on Quality Criteria

Using the scientific evaluation criteria (Table 2), the apps were rated (Table 4). The highest score was achieved by *Medisafe* (59%), followed by *MyTherapy* (56%), *Meds on time* (44%), and *Médi'rappel* (33%). For the paid apps, the highest score was achieved by *Dosecast* (40%), *Suivre ma Rx* (37%), and *Rappel de medicament* (17%). *SwissMeds* received a score of 41%.

**Table 4:** Scientific evaluation of apps.

**Category**	**No**.	**Criteria**	**Medisafe**	**MyTherapy**	**Meds on time**	**Médi'rappel**	**SwissMeds**	**Dosecast**	**Suivre ma Rx**	**Rappel de médicament**
Security and privacy	1	Password	0	1	0	0	0	0	1	0
(max five points)	2	User consent	1	1	n/a	1	1	0	0	0
	3	User consent revisable	1	1	n/a	1	1	0	0	0
	4	Erasing of user data	1	1	1	0	0	1	1	0
	5	Data collection	1	1	1	1	1	1	0	1
		Subtotal	4	5	2	3	3	2	2	1
Quality of health-related	6	Aim	1	1	1	1	n/a	1	1	1
content	7	Education	1	0	1	1	0	0	0	0
(max four points)	8	Involvement of healthcare professionals	1	0	1	1	0	0	0	0
	9	Trustworthiness, credibility, and quality of the educational information	n/a	n/a	n/a	n/a	0	0	0	0
		Subtotal	3	1	3	3	0	1	1	1
Quality of information	10	Certification	0	0	0	0	0	0	0	0
management of and	11	Content author's expertise	0	1	0	0	1	0	0	0
topic-related	12	Evaluation by target population	0	0	0	0	1	0	0	0
information (max six	13	Declaration of interest	0	0	0	0	0	0	0	0
points)	14	Sources and references	0	0	0	0	1	0	0	0
	15	Funding	0	0	0	0	0	0	0	0
		Subtotal	0	1	0	0	3	0	0	0
Functionality (max 11 points)	16	Reminders: text messages and push notifications	1	1	1	1	1	1	1	1
	17	Visual feedback	1	1	1	0	0	0	0	0
	18	Meetings	1	1	0	0	0	0	0	0
	19	Cloud/synchronization of data on different devices	1	1	0	0	0	1	0	0
	20	Access and sharing of different phone functions	n/a	n/a	n/a	n/a	1	1	1	0
	21	Patient file	1	1	0	0	0	0	0	0
	22	Manual entries and comments	1	0	0	0	1	1	1	0
	23	Gamification	0	0	0	0	0	0	0	0
	24	Support	1	1	1	0	1	1	1	0
	25	Refill	1	0	1	1	0	1	1	0
	26	Time zone adaptation	0	0	0	0	0	0	0	0
		Subtotal	8	6	4	2	4	6	5	1
Esthetics (max three	27	Flexibility	n/a	n/a	n/a	n/a	1	1	1	0
points)	28	Legibility	0	0	1	0	0	0	0	0
	29	Customization	1	1	0	0	0	1	1	1
		Subtotal	1	1	1	0	1	2	2	1
Advertising (max three	30	Advertising	0	1	1	1	1	1	1	1
points)		Subtotal	0	1	1	1	1	1	1	1
Total points (max 30 points)			16/27	15/27	11/25	9/27	12/29	12/30	11/30	5/30
			59%	56%	44%	33%	41%	40%	37%	17%

#### Patient App Evaluation Using an Online Questionnaire

Two e-patients agreed to review the top three free-of-charge apps identified by a scientific scoring of at least 35%. Their evaluation resulted in *Medisafe* (3.62) receiving the highest scores, followed by *MyTherapy* (3.37) and *Meds on time* (3.27) (Table 5).

**Table 5:** Scoring of the apps by patients (*N* = 2 of free-of-charge app testing; *N* = 10 for paid app testing).

**Apps tested by *N* patients**	**Mean score (max–min)**						
	**Engagement**	**Functionality**	**Esthetics**	**Information**	**Total Score**	**Subjective quality**	**Perceived impact**
**Free-of-charge apps**							
Medisafe (*n* = 2^a^)	3.40	4.00	3.33	3.75	3.62	3.50	n/a
MyTherapy (*n* = 2^a^)	3.60	3.25	3.33	3.33	3.37	2.00	n/a
Meds on time (*n* = 2^a^)	3.00	3.25	3.33	3.50	3.27	2.25	n/a
**Paid apps**							
Dosecast (*n* = 4)	3.50 (4.00–3.00)	4.56 (5.00–4.00)	3.75 (4.33–3.00)	3.50 (4.75–2.00)	3.83 (4.27–3.33)	3.44 (4.50–2.50)	2.17 (4.17–1.33)
SwissMeds (*n* = 4)	3.05 (4.20–1.60)	3.75 (4.75–2.75)	3.67 (4.67–2.33)	3.52 (4.75–2.33)	3.50 (4.09–2.55)	3.19 (4.25–1.25)	2.92 (5.00–1.00)
Suivre ma Rx (*n* = 1^b^)	1.40	2.75	2.67	2.50	2.33	1.00	1.00

a *The same two patients tested all selected free-of-charge apps*.

b *The second patient dropped out post-randomization but before evaluation*.

**Figure 3:**
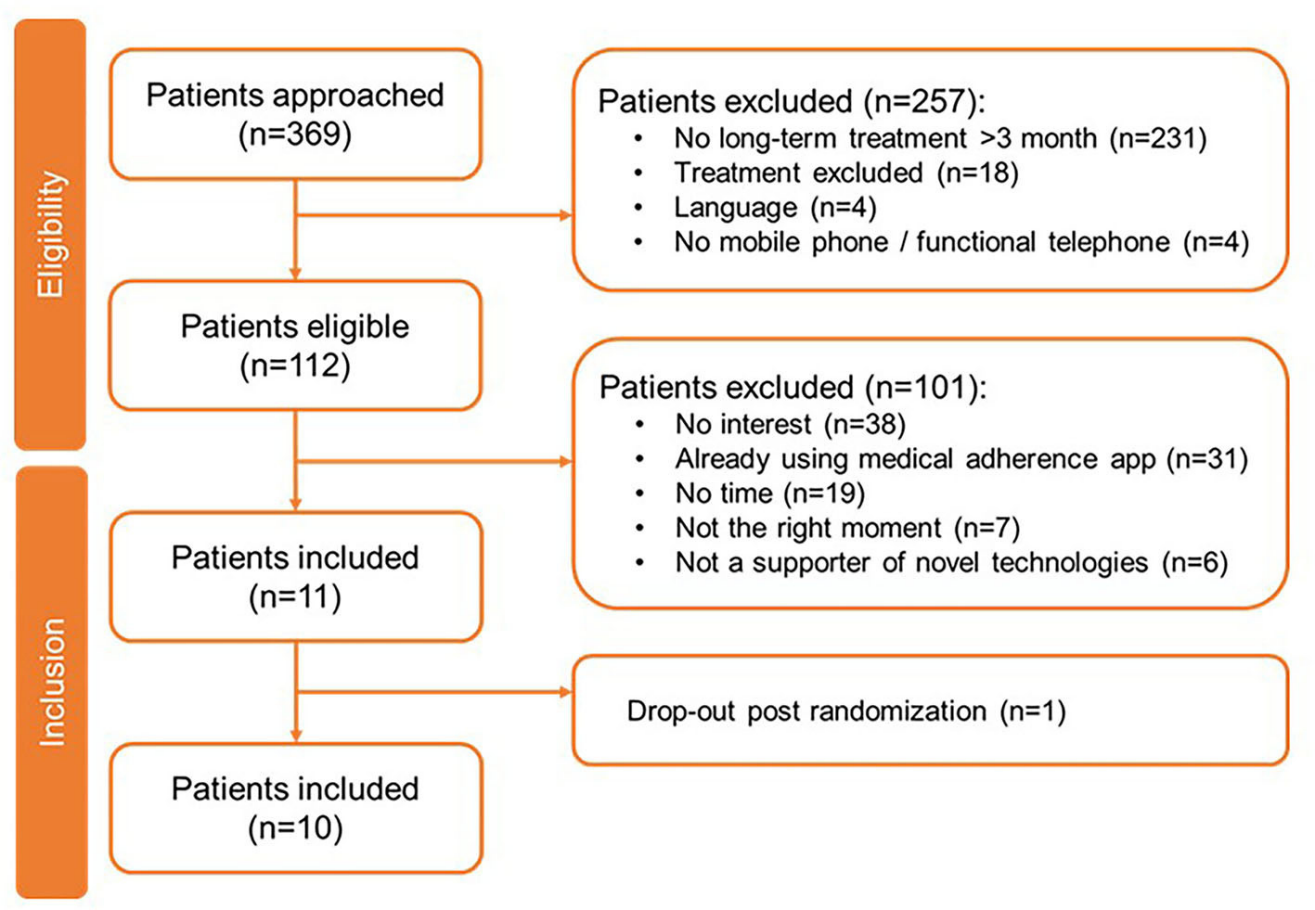
Patient inclusion flowchart for paid apps testing.

For the paid apps, 369 patients have been approached of whom 257 were excluded, primarily because they were not undergoing chronic or long-term (>3 months) treatment (*n* = 231) (Figure 3). Of the 112 eligible patients, 10 were included in this study. Their demographics are shown in Table 3. Of the 10 patients, the majority used Apple's iOS as operating system (*n* = 9) and did not have experience with health-related apps (*n* = 8). One patient on *Suivre ma Rx* dropped out post-randomization. During their evaluation, patients (*N* = 10) allocated the highest scoring on *Dosecast* (3.83), followed by *SwissMeds* (3.50) and *Suivre ma Rx* (2.33) (Table 5).

## Discussion

This study established a novel methodology to evaluate mHealth applications for medication adherence, comprising first a scientific appraisal and second an end-user appraisal. Firstly, we evaluated free-of-charge apps to get an overview of apps accessible for all. Since the results from this first evaluation were not satisfactory, we decided to pursue our study with paid apps. This method enabled the withholding of four out of 1,853 free-of-charge medication adherence apps (0.2%) and three out of 307 paid apps (0.9%). These results underline the high availably of medication apps on the market, as well as the exceptionally low number of apps which met the study inclusion criteria and that could be recommended by healthcare providers. The evaluation resulted in a wide range of average quality scores (17–57%) showing a high variability of quality and usability and a need to evaluate apps individually. Moreover, scoring levels were quite low (max 57%), suggesting significant room for improvements. The apps were evaluated through six identified evaluation domains: (i) security and privacy, (ii) quality of the health-related content, (iii) quality of the app information management, (iv) functionality, (v) user interface, and (vi) acceptability of the app. To our knowledge, this is the first study in this context conducted in this area in Switzerland. Due to the repeatability of the proposed method, this study can also be used in other environments and populations. The study is based upon a qualitative and quantitative evaluation including patient feedback allowing for the retrieval of additional information (e.g. data protection). The study was carried out with chronic disease patients who require long-term treatment and can thus benefit from medication adherence.

Security and data protection aspects of existing apps highly contribute to the low evaluation scores. Introduced on May, 25, 2018, the GDPR of the EU currently includes specific regulations for mHealth apps (28). For the US apps, e.g., *Dosecast*, US data protection laws, widely considered less strict than the European laws, apply. The free-of-charge apps score higher in the data protection category than the paid apps, similar to what Loy et al. described. This can be attributed to the fact that paid apps are more often developed by individuals than companies (40). For *Dosecast*, the terms and conditions include a statement on data collection that asserts that data is not transferred to third parties. However, a closer look reveals that data on the drugs used may be accessed by partnering companies. For end users, this ambiguity may be difficult to discern.

On the quality of the health-related content, the free-of-charge apps score higher than the paid ones as they have a clear aim and educational information and further involve healthcare professionals.

Quality of information management is handled poorly with the exception for *SwissMeds*, which was specifically programmed for this aspect. None of the apps in this research has undergone a certification process, such as CE marking, through an accredited body. In Europe, medical software, of which mHealth is a part of, is regulated through the medical device directive 2017/745/EC resulting in CE marking and will be required from 26 May 2021 onwards (26). In the USA, Food and Drug Administration (FDA) guidelines exist (41), providing information about regulatory oversight of mobile health apps that satisfy the definition of a medical device. An independent European industry label in digital health exists, called “mHealth Quality,” which evaluates apps on their medical value, ethics, privacy, security, and regulatory conformity (42). Looking at these regulations, the apps may need to be improved, which would raise the scores in this category.

None of the apps listed focuses on quality of health-related and medication adherence contents. The only app declaring a collaboration with a credible content author is MyTherapy, which has a collaboration with Charité—Universitätsmedizin Berlin (43). Conflicts of interest, financing, and sources for the content are not declared in the apps except for *SwissMeds*. This is concerning as the app *Meds on time* is part of the Japanese pharmaceutical company Daiichi Sankyo (44) and Médi'rappel is offered by the French generics pharmaceutical company Biogaran (45). The user may question these companies' interests and/or motives, as these apps are free and offer no declaration on data privacy and security. Further research and regulation are urgently needed. By supporting medication adherence, apps can be considered as intervention devices. Their constitutive components should be investigated, especially the way medication adherence is scored for patient's feedback. Moreover, the intervention content provided to the users and healthcare providers must be developed from research findings.

The functionality of medication adherence apps is determined on the basis of medication reminders. Only *Suivre ma Rx* does not provide adaptability for weekly adherence monitoring. Tailoring the app to the patient's treatment regime is essential and ideal for supporting patient's adherence (25). Some apps also provide cloud storage and sharing across several devices, allowing for information (including dedicated patient files) to be shared with other people. None of the tested apps provided a gamification mode. However, the literature has described gamification modes, for instance through trophies or leveling, as a means of improving patient engagement with apps (46).

Looking at the overall scores, *Medisafe* (59%), *MyTherapy* (56%), and *Meds on time* (44%) received the highest scores in the scientific app evaluation. In the patient evaluation, Dosecast (3.83 out of five points), Medisafe (3.62), and SwissMeds (3.50) received the highest scores. These relatively average quality scores, even among the highest scoring apps, show the difficulty that patients face in finding a high-quality app. Comparing these results to the literature, similar results have been found in a study for the Australian market, rating *Medisafe* as the best and *Dosecast* as the second best app (33). In another study in the USA, *Medisafe* was rated highest and *Dosecast* in the midfield (47). This confirms the proposed method being in accordance with the findings of other similar studies.

The uMARS does not evaluate questions regarding data privacy and security, and patients may not provide feedback on these aspects. In addition, it does not evaluate the existence and credibility of sources, which is an integral part of a scientific evaluation. Thus, uMARS should be used with end users only after a scientific, professional, and patients' app evaluation, including security and privacy aspects, as presented in this study.

The most important limitation of this study is the constant evolution of the technology and delivery of updates. Every day, some apps are removed, while others are added. Even the apps tested in this study may disappear in the future. Furthermore, apps require frequent updates, which may add new content and functionalities, improving the score in this study. Upcoming CE marking or other labeling might be possible for the apps as well. Nevertheless, this study aimed at developing a methodology that can be replicated with new apps, thus allowing comparison of the apps' performance over time.

Another limitation is the constraint to only the top 50 search results for each search term. So, apps listed outside of this constraint might be overlooked. To reduce this risk, the search was executed with several search terms reducing this risk. We mimicked patient search, hypothesizing that they would not search beyond a certain number of apps and we limited the threshold at 50. In addition, the ranking of search terms in the app stores is changed non-transparently, diminishing repeatability. Also, apps could have been excluded and not tested because of their vague description without having been tested. Apps only available on one of the two stores might have been excluded even though they may have had a high quality. However, most of the high-quality apps are offered in both stores. The evaluation of medication adherence apps is linked to the local context including the language and the app store availability (here: the French and the Swiss stores) and, thus, represents an inherent constraint of our study. However, our methodology is reproducible whatever the languages.

Scientific evaluation was based upon assigning zero or one point per category. These categories could be adapted to higher granularity of scoring to allow for weighting for some aspects (e.g. prioritizing data protection over esthetics). This needs to be investigated in future studies.

Finally, even though higher-quality applications have been identified, until today, there is no evidence that they increase medication adherence (30). There is currently no scoring system in place to analyze the app quality in relation to medication adherence measures and feedback to the patients. This is crucial and needs to be developed. Finally, clinical studies with chronic patients are needed to measure the impact of the app's long-term utilization on outcomes such as medication adherence (both implementation and persistence), patient satisfaction, and app-mediated exchange of information on medication adherence between patients and healthcare providers.

Currently, we would discourage healthcare providers from recommending these free-of-charge and paid apps because of security issues. In the long term, we underline the need of further medication app evaluations including other languages and countries, as well as larger patient groups, and hope that high-quality apps will be available in the healthcare system. SwissMeds seems promising in this regard. This will allow patients to take personal control of their own treatment through mHealth tools while supporting and strengthening patient collaboration with healthcare providers.

## Data Availability Statement

The original contributions presented in the study are included in the article/Supplementary Material, further inquiries can be directed to the corresponding author/s.

## Ethics Statement

The studies involving human participants were reviewed and approved by Ethics committee approval from the canton of Geneva, Switzerland, was obtained through application 2018-01398. The patients/participants provided their written informed consent to participate in this study.

## Author's Note

This article is a synthesis of the master theses in pharmaceutical sciences of Carla Moyano and Camille Rimaud, which have been supervised by Claudine Backes and Marie-Paule Schneider.

## Author Contributions

CBa is the main author of this manuscript and coordinated the research. This article is a synthesis of the master theses by CM and CR. CBa and MS supervised this work. CBi, an e-patient, provided crucial insight on the patient's perspective for mHealth apps. All authors contributed to the article and approved the submitted version.

## Conflict of Interest

The authors declare that the research was conducted in the absence of any commercial or financial relationships that could be construed as a potential conflict of interest.
