# Supplementary material for: Digital Medication Adherence Support: Could Healthcare Providers Recommend Mobile Health Apps?
Source: Front Med Technol. 2021 Feb 17;2:616242. doi: 10.3389/fmedt.2020.616242 (PMC8757821; doi:10.3389/fmedt.2020.616242)
Supplement: Supplementary file 1 [file Data_Sheet_1.PDF]

<u>Supplemental Material (Appendices 1-6)</u>

Appendix 1: Search terms that identified at least one of the included free-of-charge apps

Medisafe, MyTherapy, Meds on time and Médi’rappel.

Of the 4 free-of-charge apps included in this study, number (%) of the apps identified by the search terms.	**Search terms identified (n=26)**				
4 (100%)	rappel cachet<br>(English translation: tablet reminder)	medicament<br>(medication)			
3 (75%)	medication reminder<br>(-)	rappel medical<br>(medical reminder)	meds reminder<br>(-)	meds<br>(-)	rappel medicament<br>(medication reminder)
2 (50%)	medication pill reminder<br>(-)	medication alarm<br>(-)	pill reminder<br>(-)	rappel pilule<br>(pill reminder)	medication tracker<br>(-)
	medication<br>(medication)	prise pilule<br>(pill intake)	médication management<br>(medication management)	rappel medication<br>(medication reminder)	rappel comprimé<br>(tablet/pill reminder)
	Reminder<br>(-)	prise medicament<br>(medication intake)			
1 (25%)	Dose<br>(dose)	Dosage<br>(dosage)	rappel traitement<br>(treatment reminder)	rappel traitement medical<br>(medical treatment reminder)	rx
	prise traitement<br>(intake reminder)	prise comprimé<br>(intake tablet/pill)			

Appendix 2: Online questionnaire and list sent to e-patients to identify free-orf-charge medication adherence apps (in French).

Utilisation de mHealth

MHealth est l'abréviation de mobile health. Ce sont des applications de santé que l'on emploie sur les smartphones ou d'autres appareils mobiles. Un exemple de mHealth est une app de gestion de la prise de médicaments par le patient.

1. **Quel est le système d'exploitation de votre smartphone ? ***

   Une seule réponse possible.

   - iOs de Apple
   - Android de Google
   - Windows phone de Microsoft
   - Autre : ______

2. **Utilisez-vous des mHealth ? ***

   Une seule réponse possible.

   - Oui
   - Non

3. **Si vous utilisez des mHealth, quelle est votre fréquence d'utilisation ?**

   Une seule réponse possible.

   - 1x par jour
   - 2x par jour
   - 3x par jour
   - Plus de 3x par jour
   - 3x par semaine (environ 1 jour sur 2)
   - 1x par semaine
   - 1x toutes les deux semaines
   - 1x par mois
   - Moins de 1x par mois
   - Je n'utilise pas de mHealth

4. **Si vous devez choisir une app en lien avec votre traitement , en quelle langue la choisissez-vous ? ***

   Plusieurs réponses possibles.

   - [ ] Français
   - [ ] Anglais
   - [ ] Autre : ______

5. **Quelles autres technologies utilisez-vous dans le cadre de votre maladie ? ***

   Exemple : forums, sites internet particuliers, blog de santé, etc. Si vous n'en utilisez pas vous pouvez écrire "rien".

Termes de recherche

Nous nous intéressons maintenant, aux termes de recherche ou mots-clés que vous introduisez dans l'Apple Store, le Google play ou un autre store pour smartphone lorsque vous cherchez des mHealth.

6. **Si vous deviez rechercher une app pour soutenir la prise de votre traitement chronique au quotidien, quels sont les termes de recherche que vous utiliseriez ? ***
Exemple de terme de recherche : rappel de médicament

7. **Pouvez-vous citer une ou plusieurs apps que vous utilisez pour soutenir la prise de votre traitement ?**

Termes de recherche

8. **Voici les termes auxquels nous avons pensé. Merci de passer en revue cette liste et de cocher tousles mots-clés que vous utiliseriez ***
Attention certains termes sont en anglais et d'autres en français
Plusieurs réponses possibles.

- [ ] medication reminder
- [ ] medication pill reminder
- [ ] meds reminder
- [ ] pill reminder
- [ ] rappel médication
- [ ] rappel médicament
- [ ] rappel traitement
- [ ] rappel traitement médical
- [ ] rappel médocs
- [ ] rappel pilule
- [ ] rappel comprimé
- [ ] rappel cachet
- [ ] medication tracker
- [ ] médication management
- [ ] management médicament
- [ ] management traitement
- [ ] management traitement médical
- [ ] gestion des médicaments
- [ ] gestion traitement

- [ ] gestion traitement médical
- [ ] gestion médocs
- [ ] rx
- [ ] médication
- [ ] médicament
- [ ] meds
- [ ] médocs
- [ ] traitement
- [ ] traitement médical
- [ ] adherence
- [ ] adhésion
- [ ] compliance
- [ ] observance
- [ ] observance traitement
- [ ] dose
- [ ] dosage
- [ ] posologie
- [ ] drug
- [ ] drogue
- [ ] remind
- [ ] reminder
- [ ] système mémoire
- [ ] mémento
- [ ] pense-bête
- [ ] système rappel
- [ ] aide-mémoire
- [ ] système mémorisation
- [ ] therapy
- [ ] thérapie
- [ ] thérapeutique
- [ ] medication alarm
- [ ] alarme médicament
- [ ] alarme traitement
- [ ] prise traitement
- [ ] prise médicament
- [ ] prise traitement médical
- [ ] prise pilule
- [ ] prise comprimé

Questions socio-démographiques

Nous espérons avoir une population de patients aussi diverse que possible. Ce qui permettrait de représenter la population de patients chroniques. Les questions ci-dessous nous permettront de savoir si tel est réellement le cas.

9. **Sexe** *
   Une seule réponse possible.
   - ( ) Homme
   - ( ) Femme

10. **Âge** *
    Une seule réponse possible.
    - ( ) 18 - 20 ans
    - ( ) 20 - 30 ans
    - ( ) 30 - 40 ans
    - ( ) 40 - 50 ans
    - ( ) 50 - 60 ans
    - ( ) 60 - 70 ans
    - ( ) 70 ans et +

11. **Langue maternelle** *
    Plusieurs réponses possibles.
    - [ ] Français
    - [ ] Anglais
    - [ ] Autre : ______________________

12. **Prenenz-vous des médicaments de façon chronique ?** *
    Une seule réponse possible.
    - ( ) Oui
    - ( ) Non

13. **Si vous prenez des médicaments de façon chronique, de quel type de médicaments s'agit-il ?**

Médicament pour :

Plusieurs réponses possibles.

- [ ] Maladie cardio-vasculaire
- [ ] Maladies respiratoire
- [ ] Maladie rénale
- [ ] Maladie digestive
- [ ] Maladie dermatologique
- [ ] Maladies infectieuse
- [ ] Maladie neurodégénérative
- [ ] Maladie psychiatriques
- [ ] Maladie génétique
- [ ] Maladie et trouble oculaire
- [ ] Maladies rare
- [ ] Cancer
- [ ] Douleurs chroniques
- [ ] Autre : ____________________

14. **Depuis combien de temps prenez-vous vos médicaments de façon chronique ?** *

Une seule réponse possible.

- ( ) Moins d'une année
- ( ) 1 à 2 ans
- ( ) 2 à 5 ans
- ( ) 5 à 10 ans
- ( ) Plus de 10 ans
- ( ) Pas de médicaments

15. **Dans quelle catégorie professionnelle vous situez-vous ?** *

Une seule réponse possible.

- Dirigeants, cadres supérieurs, cadres de direction
- Professions intellectuelles et scientifiques (ingénieurs, médecins, professeurs, avocats, etc.)
- Professions intermédiaires (techniciens, infirmiers, comptables, inspecteurs de police, etc.)
- Employés de type administratif (secrétaires, standardiste, guichetiers, etc.)
- Personnel des services et de la vente (cuisiniers, serveurs, coiffeurs, pompiers, guides, vendeurs, etc.)
- Agriculteurs et ouvriers qualifiés de l'agriculture, de la sylviculture et de la pêche
- Métiers qualifiés de l'industrie et de l'artisanat (maçons, charpentiers, couvreurs, plâtriers, potiers, orfèvres, bouchers, boulangers, ébénistes, couturiers, etc.)
- Conducteurs de machine et de robots industriels, grutiers, chauffeurs de taxi, pilotes de locomotive, etc.
- Ouvriers et employés non qualifiés (manutentionnaires, éboueurs, livreurs, aides de ménage, vendeurs ambulants, etc.)
- Professions militaires
- Autre : ____________________

16. **Adresse mail**

Dans une deuxième étape de ce projet, nous souhaiterions tester 2 ou 3 applications de santé avec l'aide de patients volontaires. Chaque application serait testée pendant 10 jours durant le mois de mai 2017. L'objectif de ce travail est de mettre à disposition des pharmaciens d'officine une sélection d'applications répondant aux mieux aux besoins des patients chroniques de Suisse romande en 2017 afin qu'ils puissent les leur proposer. Si vous souhaitez participer à la suite de ce projet, nous vous serions reconnaissantes de nous communiquer votre adresse mail ci-dessous. Bien entendu, votre adresse email ne sera utilisée que dans le cadre de la 2ème étape de cette étude, elle ne sera en aucun cas transmise à des tiers et nous certifions qu'elle sera détruite au terme de l'analyse des données à la fin juin 2017.

____

____

____

____

____

Commentaires, remarques, questions

Vous pouvez également me contacter à mon adresse mail pour toutes autres questions ou remarques : carla.moyano@etu.unige.ch

17.

____

____

____

____

____

Appendix 3: Online questionnaire based upon uMARS used for patient app evaluation (in French).

Application

Nous vous serions reconnaissantes de nous communiquer votre adresse mail ou votre nom ci-dessous afin que nous puissions savoir de qui provient la réponse.

Bien entendu, votre adresse email ne sera utilisée que dans le cadre de cette étude, elle ne sera en aucun cas transmise à des tiers et nous certifions qu'elle sera détruite au terme de l'analyse des données à la fin juin 2017.

1. **Adresse mail ou nom** *

2. **Quelle application avez-vous testée ?** *
   Une seule réponse possible.
   - Medisafe
   - MyTherapy
   - Médi'rappel
   - Meds on Time

Section A : Engageant

Amusant, intéressant, personnalisable, interactif (envoie des alertes, messages, reminder, feedback, permet le partage

3. **1. Divertissement : est-ce que l'application est divertissante/sympa à utiliser ? Est-ce qu'elle contient des composantes qui la rendent plus divertissante que d'autres apps similaires.** *
   Une seule réponse possible.
   - Ennuyeuse, pas amusante ou divertissante du tout
   - Généralement ennuyeuse
   - OK, assez amusante pour divertir l'utilisateur pendant un bref moment (<5 minutes)
   - Modérément amusante et divertissante, divertirait l'utilisateur pendant un certain temps (5 à 10 minutes au total)
   - Très divertissante et amusante, stimulerait l'utilisation répétée de l'application

4. **2. Intérêt : est-ce que l'application est intéressante à utiliser ? Est-ce qu'elle présente ses informations de manière intéressante comparée à d'autres applications similaires?** *
   Une seule réponse possible.
   - Pas intéressante du tout
   - Généralement pas intéressante
   - OK, ni intéressante, ni pas intéressante ; intéresserait l'utilisateur pendant un bref moment (<5 minutes)
   - Modérément intéressante ; intéresserait l'utilisateur pendant un certain temps (5-10 minutes au total)
   - Très intéressante, l'utilisateur pourrait l'utiliser de façon répétée

5. **3. Personnalisable : est-ce qu'elle vous permet de personnaliser/customiser les paramètres et les préférences que vous aimeriez (par exemple, le son, le contenu et les notifications)?** *
   Une seule réponse possible.
   - N'autorise aucune personnalisation ou requière de saisir les paramètres à chaque fois
   - Permet peu de personnalisation ce qui limite les fonctions de l'application
   - Personnalisation basique pour fonctionner de manière adéquate
   - Permet de nombreuses options de personnalisation
   - Permet de personnaliser complètement les caractéristiques / préférences de l'utilisateur, se souvient de tous les paramètres

6. **4. Interactivité : est-ce que l'app permet à l'utilisateur d'entrer des données, est-ce qu'elle fournit un feedback, est-ce qu'elle donne des alertes (reminders/rappels, notifications, etc.) ? ***
   Une seule réponse possible.

   - ( ) Aucune fonction interactive et/ou aucune réponse aux entrées de données de l'utilisateur
   - ( ) Quelques fonctions interactives, mais pas suffisamment, ce qui limite les fonctions de l'application
   - ( ) Fonctions interactives basiques permettant de fonctionner de manière adéquate
   - ( ) Offre une variété de fonctions interactives, de feedback et/ou permet à l'utilisateur d'entrer des données
   - ( ) Très haut niveau d'interactivité grâce aux fonctions interactives, aux feedback et/ou aux options d'entrée de données des utilisateurs

7. **5. Groupe cible : est-ce que le contenu de l'application (information visuelle, langage, design) est approprié au public à qui l'app est destinée ? ***
   Une seule réponse possible.

   - ( ) Complètement inapproprié, pas clair ou confus
   - ( ) Généralement inapproprié, peu clair ou confus
   - ( ) Acceptable mais pas spécifiquement conçu pour le public cible. Peut parfois être inapproprié / peu clair / confus
   - ( ) Conçu pour le public cible mais avec des problèmes mineurs
   - ( ) Conçu spécifiquement pour le public cible, aucun problème n'a été trouvé

8. **Vos commentaires**

   ____
   ____
   ____
   ____
   ____

Section B – Fonctionnalité

Fonctionnement de l'application, facile à apprendre, navigation, flux/parcours logique, design gestuel de l'application

9. **6. Performance : avec quelle précision/vitesse est-ce que les fonctions de l'application et les éléments (boutons/icônes/menus) fonctionnent ? ***

Une seule réponse possible.

- L'application ne fonctionne pas; pas de réponse/ réponse insuffisante / réponse inexacte (par exemple crash / bugs / fonctionnalités qui ne fonctionnent pas, etc.)
- Certaines fonctions fonctionnent, mais de façon lente ou contiennent des problèmes techniques majeurs
- L'application fonctionne globalement. Certains problèmes techniques doivent être réparés, ou elle est lente à certains moments
- Fonctionnelle la plupart du temps avec des problèmes mineurs / négligeables
- Réponse parfaite/opportune ; aucun bug technique trouvé, ou contient un indicateur de "temps de chargement restant" (si pertinent)

10. **7. Facilité d'utilisation : avec quelle facilité est-il possible d'apprendre à utiliser l'application ; quel est le niveau de clarté des icônes/boutons du menu et des instructions ? ***

Une seule réponse possible.

- Pas ou peu d'instructions; le nom/icône des menus génère de la confusion; est compliqué
- Prend beaucoup de temps ou d'efforts
- Prend un peu de temps ou d'effort
- Facile à apprendre (ou les instructions sont claires)
- Capable d'utiliser l'application immédiatement; intuitif; simple (aucune instruction nécessaire)

11. **8. Navigation : est-ce que le déplacement entre les écrans/pages est logique ? Est-ce que tous les liens vers les écrans/pages nécessaires sont présents ? ***

Une seule réponse possible.

- Aucune connexion logique du tout entre les écrans / la navigation est difficile
- Compréhensible après beaucoup de temps / d'effort
- Compréhensible après un peu de temps / d'effort
- Facile à comprendre / à naviguer
- La navigation entre les écrans est parfaitement logique, facile, claire et intuitive, et / ou comporte des raccourcis

12. **9. Design gestuel : est-ce que les interactions (toucher, glisser, zoomer/dézoomer, défiler) sont cohérentes et intuitives dans tous les écrans/pages/menus ? ***

Une seule réponse possible.

- Complètement incohérent / génère toujours de la confusion
- Souvent incohérent / génère souvent de la confusion
- OK mais avec certaines incohérences ou génère par moment de la confusion
- Généralement cohérent / intuitif avec des problèmes négligeables
- Parfaitement cohérent et intuitif

13. **Vos commentaires**

Section C – Esthétique

Design des graphismes, attractivité visuelle, cohérence des couleurs et des styles

14. **10. Mise en page : est-ce que la disposition et la taille des boutons/touches/icônes/menus/contenu de l'écran sont appropriées ? ***

Une seule réponse possible.

- Très mauvais design, chaotique, certaines options sont impossibles à sélectionner, à localiser, à voir ou lire
- Mauvais design, aléatoire, pas clair, certaines options sont difficiles à sélectionner / localiser / voir / lire
- Satisfaisant, peu de problèmes avec la sélection / la localisation / la vue / la lecture des éléments
- Généralement clair, permet de sélectionner / localiser / voir / lire les éléments
- Professionnel, simple, clair, ordonné, organisé logiquement

15. **11. Graphisme : quel est le niveau de qualité/résolution des graphiques utilisés pour les boutons/icones/menus/contenu ? ***

Une seule réponse possible.

- Les graphiques sont amateurs, design visuel très pauvre, disproportionné, stylistiquement incohérent
- Graphiques de mauvaise qualité / basse résolution; design visuel de mauvaise qualité, disproportionné
- Graphiques et design visuel de qualité modérée (style généralement cohérent)
- Graphiques et design visuel de haute qualité / résolution, en grande partie proportionné, style cohérent
- Graphiques et design visuel de très haute qualité / résolution, proportionné, style toujours cohérent

16. **12. Attractivité visuelle : est-ce que l'application est visuellement belle ? ***

Une seule réponse possible.

- Laide, désagréable à regarder, design pauvre, couleurs incompatibles
- Mauvaise, design pauvre, mauvaise utilisation de la couleur, visuellement ennuyeuse
- Ordinaire, ni agréable, ni désagréable
- Agréable, graphiques harmonieux, conçue de façon cohérente et professionnelle
- Belle, très attractive, mémorable, se distingue; l'utilisation de la couleur améliore les fonctions / menus de l'application

17. **Vos commentaires**

Section D – Information

Contient de l'information de haute qualité (exemple : texte, feedback, mesures, références) provenant de sources fiables. N/A = non applicable

18. **13. Qualité de l'information : est-ce que le contenu de l'application est correct, bien écrit, et adapté à l'objectif/thématique de l'application ?** *

Une seule réponse possible.

- N/A Il n'y a aucune information dans l'application
- Sans pertinence / inappropriée / incohérente / incorrecte
- Pauvre. A peine pertinente / appropriée / cohérente / peut être incorrecte
- Modérément pertinente / appropriée / cohérente / et semble correcte
- Pertinente / appropriée / cohérente / correcte
- Très pertinente, appropriée, cohérente et correcte

19. **14. Quantité d'informations : est-ce que la quantité d'informations est complète tout en restant concise ?** *

Une seule réponse possible.

- N / A Il n'y a aucune information dans l'application
- Minimale ou excessive
- Insuffisante ou possiblement excessive
- OK mais ni complète, ni concise
- Offre une large gamme d'informations, présente quelques lacunes ou détails inutiles; ou n'a pas de liens vers des informations et ressources complémentaires
- Complète et concise ; contient des liens vers des informations et ressources complémentaires

20. **15. Information visuelle : est-ce que l'explication visuelle des concepts (au travers de schémas/graphiques/images/vidéos, etc.) est claire, logique, et correcte ?** *

Une seule réponse possible.

- N / A Il n'y a pas d'information visuelle dans l'application (par exemple, elle ne contient que de l'audio ou du texte)
- Complètement non claire / confuse / fausse ou information nécessaire manquante
- Généralement peu claire / confuse / fausse
- OK mais souvent peu claire / confuse / fausse
- Souvent claire / logique / correcte avec des problèmes négligeables
- Parfaitement claire / logique / correcte

21. **16. Crédibilité : est-ce que l'information au sein de l'app semble provenir d'une source crédible ? ***

Une seule réponse possible.

- N / A Il n'y a aucune information dans l'application
- Source suspecte
- Manque de crédibilité
- Pas suspecte, mais la légitimité de la source n'est pas claire
- Provient probablement d'une source légitime
- Provient avec certitude d'une source légitime / spécialisée

22. **Vos commentaires**

____

____

____

____

____

Section E - Partie subjective

23. **18. Est-ce que vous recommanderiez cette application à des personnes qui pourraient en tirer un bénéfice ? ***

Une seule réponse possible.

- Je ne recommanderais cette app à personne
- Je recommanderais cette app à peu de personnes
- Je recommanderais cette app à quelques personnes
- Je recommanderais cette app à beaucoup de personnes
- Je recommanderais cette app à tout le monde

24. **19. Combien de fois pensez-vous que vous pourriez utiliser cette application dans les 12 prochains mois si elle vous était pertinente ? ***

Une seule réponse possible.

- Jamais
- 1 – 2 fois
- 3 – 10 fois
- 10 – 50 fois
- > 50 fois

25. **20. Paieriez-vous pour cette application ? ***

Une seule réponse possible.

- 1. Non, pas du tout
- 2. Peut-être
- 3. Oui

26. **21. Quelle note globale attribueriez-vous à cette application ? (1= la pire app que je n'aie jamais utilisée ; 5= la meilleure app que je n'aie jamais utilisée) ***

Une seule réponse possible.

- 1
- 2
- 3
- 4
- 5

27. **Vos commentaires**

Félicitations, vous avez répondu à toutes les questions ! Un grand Merci !

Nous vous remercions chaleureusement d'avoir consacré du temps à cette étude. Dès la réception de vos réponses, nous vous contacterons pour le test d'une autre application.

Nous restons à votre entière disposition pour toutes questions et nous vous remercions à nouveau pour votre précieuse collaboration.

Carla Moyano, 1ère année Master en Sciences Pharmaceutiques, Université de Genève, Suisse (carla.moyano@etu.unige.ch) et atteignable aux heures de bureau au 022 379 55 95
Marie Paule Schneider, Pharmacie de la Policlinique Médicale Universitaire (PMU) à Lausanne, Suisse (Marie-Paule.Schneider@hospvd.ch)
Claudine Backes, Collaboratrice scientifique à la PMU à Lausanne, Suisse, (Claudine.Backes@chuv.ch)

Fourni par
Google Forms

Appendix 4: Patient recruitment inclusion and exclusion criteria for patient app evaluation testing.

Inclusion criteria (n=5)	Exclusion criteria (n=4)
**At least 18 years of age**	High-maintenance patients needing everyday healthcare
**At least one chronic disease treatment (for at least 3 month)**	Already using a medication adherence app
**Owning a smartphone (iOS or Android)**	Use medication with narrow therapeutic index
**Speak and read French**	Only use of oral contraceptives
**Accepting to test 1-2 apps**	

Appendix 5: Flowchart for the randomized e-patient testing.

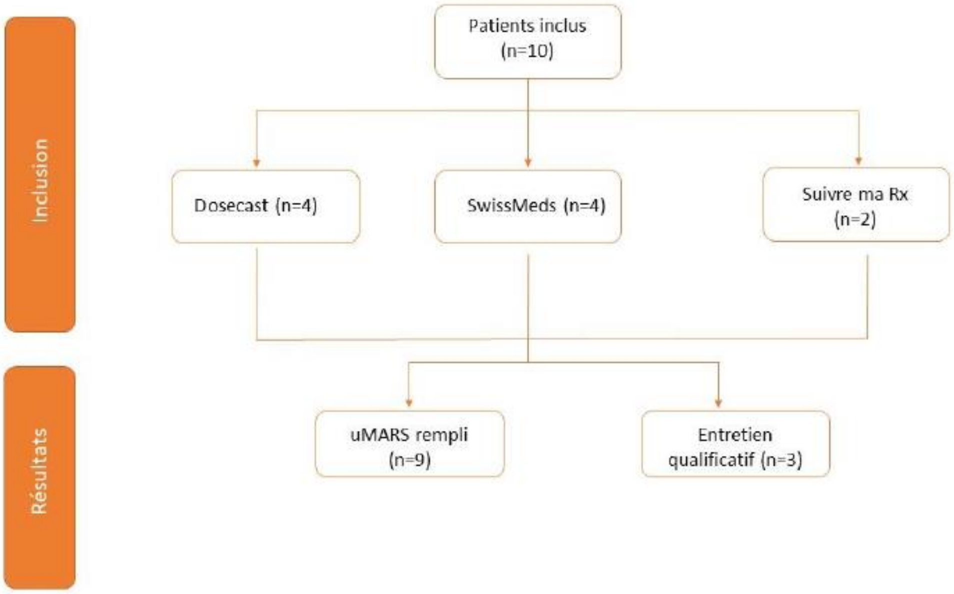

Appendix 6: Search terms identified by experts for medication adherence apps (English and French, n=82).

Search terms (n=82)			
rappel médicament	management médicament	adhésion	medication reminder
rappel traitement	management traitement	adhésion thérapeutique	meds reminder
rappel comprimé	management traitement médical	compliance du malade	meds
rappel médocs	management médocs	compliance du patient	médication management
rappel cachet	gestion des médicaments	compliance de l'utilisateur	médication
rappel pilule	gestion traitement	coopération du malade	pill reminder
rappel médication	gestion traitement médical	coopération du patient	medication alarm
rappel médical	gestion traitements médicamenteux	observance	medication pill reminder
rappel traitement médical	gestion médocs	observance prescription	reminder
prise médicament	gestion des pharmacothérapies	observance traitement	adherence
prise pilule	système rappel	compliance	remind
prise comprimé	système mémorisation	assiduité au traitement	medication tracker
prise traitement	système d'aide-mémoire	médicament	therapy
prise traitement médical	système mémoire	médocs	dosage
prise médocs	mémento	traitement	dose
prise cachet	aide-mémoire	traitement par médicament	posologie
donner des médicaments	pense-bête	traitement médical	préparation pharmaceutique
traqueur médicaments	alarme médicament	traitement médicamenteux	thérapeutique médicamenteuse
tracker médicament	alarme traitement	traitement pharmacologique	
tracker traitement	rx	thérapie	
tracker traitement médical	narcotique	thérapeutique	
tracker médocs			
